# The Management of Dysfunctional Gallbladder Disease and the Role of Laparoscopic Cholecystectomy on Symptom Improvement: A Retrospective Cohort Study

**DOI:** 10.7759/cureus.64726

**Published:** 2024-07-17

**Authors:** Mohamed Y Abuahmed, Ali Wuheb, George Eskandar, Rajeev Parameswaran, Andrew Masters, Muhammad Javed, Jeremy Wilson, Conor Magee

**Affiliations:** 1 Upper GI Surgery, Wirral University Teaching Hospital NHS Foundation Trust, Birkenhead, GBR; 2 General Surgery, Wirral University Teaching Hospital NHS Foundation Trust, Birkenhead, GBR

**Keywords:** hypofunctional gallbladder, hyperfunctional gallbladder, laparoscopic cholecystectomy, biliary dyskinesia, hida scan

## Abstract

Background

Biliary dyskinesia (BD) is a disorder characterised by abdominal pain of biliary origin (i.e., sudden steady pain at the right upper quadrant of the abdomen or the epigastrium, the absence of gallstones on ultrasound (US)), and a decreased gallbladder ejection fraction (GBEF) on a cholecystokinin-cholescintigraphy hepatobiliary iminodiacetic acid (CCK-HIDA) scan. Patients experiencing symptoms suggestive of biliary obstruction, but lacking gallstones, yet exhibiting abnormal gallbladder emptying, may find therapeutic benefit from laparoscopic cholecystectomy. Common symptoms include recurrent, intense, and enduring pain, often exacerbated by fatty food consumption, localised in the upper right quadrant or epigastric region. This pain may radiate to the back or shoulder, persisting for at least 30 minutes but not exceeding several hours, and it is sometimes accompanied by nausea and vomiting. Abnormal gallbladder emptying is typically indicated by a GBEF below 35% on cholescintigraphy following cholecystokinin administration.

Objective

This study represents a single-centric review focusing on 88 patients over a five-year period who presented with features of dysfunctional gallbladder and underwent cholescintigraphy. The primary aim was to identify whether there is any role for laparoscopic cholecystectomy in symptom improvement among these patients.

Methods

This was a retrospective cohort study involving data collection using electronic medical records. Eighty-eight patients who underwent the HIDA scan between January 2019 and December 2023 at Wirral University Teaching Hospital NHS Foundation Trust (WUTH) were identified and separated into two groups, either hypofunctioning gallbladder (EF<35% ) or hyperfunctioning gallbladder (EF>80%). Normal HIDA scan patients (EF between 35%-80%) were excluded. The frequency of laparoscopic cholecystectomy and subsequent symptom improvement were recorded.

Results

Fifty-one patients were diagnosed with gallbladder dyskinesia (BD). Of these, 36 patients (30 females, mean age 43) were diagnosed with hypofunctional gallbladder (EF<35%), where 17 patients underwent laparoscopic cholecystectomy, resulting in symptom improvement in 10 patients (58.8%). Conversely, 15 patients were diagnosed with hyperfunctional gallbladder (13 females, mean age 48.6). Only two patients (13%) underwent laparoscopic cholecystectomy with 100% symptom improvement in both patients.

Conclusions

In conclusion, our retrospective study highlights the significance of the HIDA scan in identifying gallbladder hypofunction among patients presenting with biliary symptoms. The findings establish the efficacy of laparoscopic cholecystectomy as a management approach, with a notable proportion of patients experiencing symptom improvement (58.8%). These results contribute to our understanding of biliary dysfunction management and emphasise the importance of individualised treatment strategies for optimal patient outcomes. Further, randomised controlled trials (RCTs) are warranted to validate these findings and explore additional factors influencing symptom resolution in this patient population.

## Introduction

Dysfunctional gallbladder can be defined as abdominal symptoms attributable to the biliary tract in association with abnormal gallbladder function in the absence of gallstones. Rome Foundation has developed ROME IV criteria to identify and diagnose biliary dyskinesia (BD) [1,2]. Diagnosis begins by identifying biliary-type pain, which is characterised by being located in the epigastrium and/or right upper quadrant. The pain builds up to a steady level, lasts 30 minutes or longer, occurs at different intervals, and is severe enough to interrupt daily activities. Moreover, it is neither significantly related to bowel movements nor significantly relieved by postural change or acid suppression. This type of pain may be associated with nausea and vomiting, radiation to the back and/or right infra subscapular region, and severe enough to wake the patient from sleep.

Furthermore, Rome IV set the criteria for functional gallbladder disorder, which are the same criteria for biliary pain, in addition to the absence of gallstones or other structural pathology and in the presence of supportive criteria in the form of low ejection fraction (EF) on technetium-99 hepatobiliary scintigraphy with cholecystokinin (CCK) stimulation hepatobiliary iminodiacetic acid scan (i.e., CCK-Tc-HIDA scan) abnormal gallbladder emptying is usually defined as a gallbladder EF (GBEF) of less than 35% and normal liver enzymes, conjugated bilirubin, and amylase/lipase [3].

The underlying mechanisms of BD remain uncertain. One proposed theory suggests that a constricted cystic duct could hinder the full emptying of the gallbladder, potentially resulting in persistent cholecystitis and the associated pain [4].

The CCK-Tc-HIDA scan has been developed as a means to objectively assess gallbladder contractility function by measuring GBEF. The procedure requires a large field-of-view gamma camera and a low-energy all-purpose collimator. The first hour of the exam is standard regardless of the indication. The patient lies supine on a table. TC-99 is rapidly infused, and a computer starts collecting images. For the first minute, 60 one-second frames are acquired, followed by 60 one-minute frames. At one hour, left anterior oblique and right lateral views are obtained to differentiate signal from the common bile duct, gallbladder, and duodenum. The left anterior oblique view shifts the gallbladder anteriorly and the common bile duct and duodenum posteriorly. When the gallbladder fills with radioactive material, sincalide (i.e., a cholecystokinetic drug) at 0.02 μg/kg is infused at a slow constant rate over an additional 60 minutes with images acquired over the same period [5,6]. GBEF can then be calculated as GBEF%= GB counts (maximum) - GB counts (minimum) / GB counts maximum.

Patients with symptoms suggestive of biliary obstruction but lacking gallstones but showing abnormal gallbladder emptying may benefit from laparoscopic cholecystectomy. Studies have shown that a significant proportion of individuals with BD experience improvement post-cholecystectomy, about 70% [2,7,8]. However, in a fair proportion of cases, about 30% will not benefit from laparoscopic cholecystectomy and will continue to suffer from the same symptoms even after surgery.

According to the Society of American Gastrointestinal and Endoscopic Surgeons (SAGES) guidelines on gallbladder dyskinesia, severe symptoms, a substantially reduced GBEF (<14%), and symptom reproduction during cholecystokinin administration on HIDA scan are strong indicators for symptom relief following cholecystectomy for dyskinetic gallbladder [9]. Notably, gallstones are found in only about 10%-12% of specimens from patients undergoing cholecystectomy for BD, indicating a considerable false negative rate for gallbladder US in this specific patient population [10].

In patients with impaired gallbladder function and gallstones, symptoms may not always be attributed to bile stagnation in a dysfunctional gallbladder, despite its role in gallstone formation. Gallbladder dysfunction is often overlooked as a contributing factor to gallstone development, although it plays a significant role [11,12]. Factors such as metabolic disorders, obesity, genetic traits, and environmental influences such as rapid weight loss and physical inactivity contribute to gallstone disease [13,14]. Additionally, post-bariatric surgery, young patients with preoperative pain syndromes and asymptomatic gallstones are at higher risk for symptomatic gallstone disease [15]. Therefore, a comprehensive understanding of the multifactorial nature of gallstone disease, including gallbladder dysfunction, is crucial for effective management and prevention strategies [16].

This study aimed to identify the role of laparoscopic cholecystectomy in symptom improvement among HIDA scan-proven dysfunctional gallbladder patients and whether the other modalities of medical treatment act as an alternative for surgery in the improvement of biliary-type pain.

This article is accepted for oral presentation at the 2024 11th World Society of Emergency Surgery (WSES) Annual Congress in Rhodes, Greece, on June 28, 2024.

## Materials and methods

This was an observational study conducted in a retrospective cohort study designed to assess the efficiency of laparoscopic cholecystectomy in symptom improvement for dysfunctional gallbladder disease, together with the role of medical treatment as an alternative to surgery in case of patient refusal or consultant recommendation.

We included all patients who were diagnosed as having dysfunctional gallbladders using a HIDA scan, during the period between January 2019 and December 2023 at Wirral University Teaching Hospitals, at Arrowe Park Hospital. Most of these patients were females (43 patients) whose median age was 39. Patients were then divided into two groups based on their GBEF% on the HIDA scan: hypofunctional gallbladder whose gallbladder EF is less than 35% on the HIDA scan, and hyperfunctional gallbladder whose gallbladder EF is more than 80% on the HIDA scan. Excluded were the patients with normal GBEF (i.e., GBEF% between 35% and 80%).

**Figure 1:**
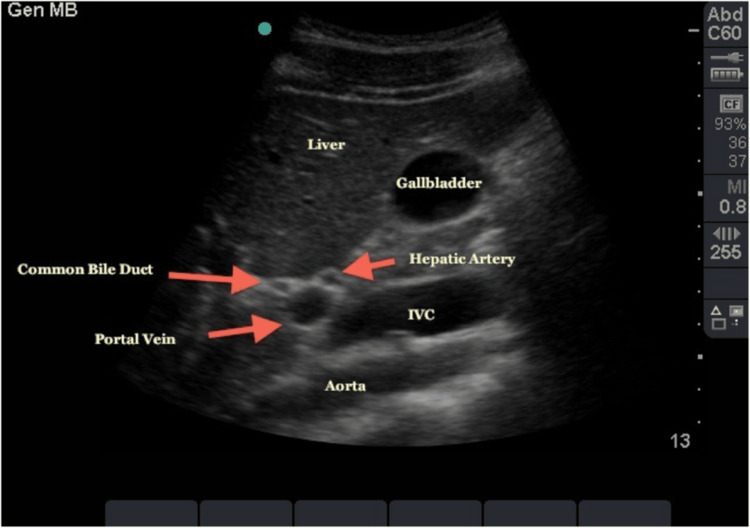
Ultrasound scan of the biliary system showing normal gallbladder and bile ducts. IVC: Inferior vena cava Source: http://www.em.emory.edu/ultrasound/Image

All patients were seen by an upper gastrointestinal consultant in the outpatient (OP) clinic. All patients had biliary-type pain according to ROME IV criteria. All patients had their liver function test (LFTs) withdrawn together with their amylase level. All patients underwent a US scan (USS) of the abdomen to exclude the presence of gallstones or dilated bile ducts (Figure 1).

After the exclusion of gallstones, patients were then referred to undergo a HIDA scan to assess the GBEF%. After the diagnosis of dysfunctional gallbladder, all patients were offered laparoscopic cholecystectomy, with the emphasis that 70% of the patients with dysfunctional gallbladder experience symptom improvement, while highlighting all the possible complications of the procedure including bile duct injury, obstructive jaundice bleeding, or surgical site infection.

Data were collected using electronic data records in Millennium Cerner software used at the Wirral University Teaching Hospital, which is a multimillion Electronic Health Records (EHR) platform that supports an enterprise-wide view of patient care and the point at which care was delivered in both acute inpatient and outpatient settings.

Data analysis was done using the Jamovi statistical analysis program (https://www.jamovi.org/) to calculate the frequency and percentage of patients who underwent laparoscopic cholecystectomy among hypofunctional gallbladder vs hyperfunctional gallbladder patients together with symptom improvement in both groups. Moreover, the frequency and percentage of patients who had alternative medical treatment and the rate of symptom improvement were analysed using the same statistical analysis program.

This study was approved by the local clinical governance committee of the hospital. No ethical concerns were raised in this study, and no ethical approval was needed because it was conducted as an audit using electronic data collection.

## Results

During the period spanning 2019-2023, there were a total of 88 patients who underwent HIDA scan for gallbladder at a single institution. Among them, 51 patients had dysfunctional gallbladder, and 36 patients were excluded for having a normal functioning gallbladder. Additionally, one patient, who had a low EF% of 19% on the HIDA scan but concomitantly had positive gallstones on the USS abdomen, was also excluded. All patients underwent a HIDA scan according to the guidelines mentioned above to calculate their GBEF.

**Figure 2:**
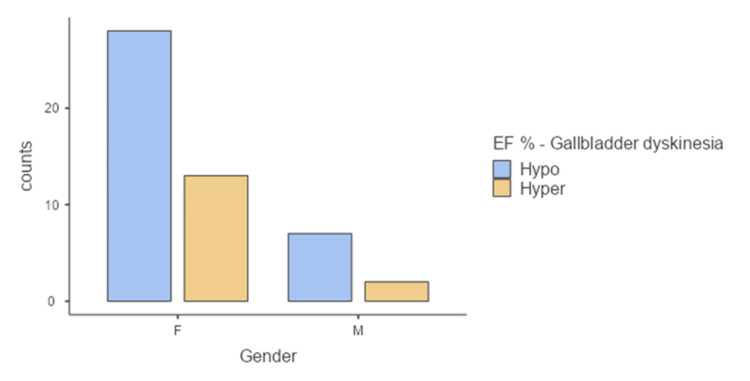
Comparison between two groups according to gender. F: female; M: male; Hypo: hypofunctional gallbladder; Hyper: hyperfunctional gallbladder; EF%: ejection fraction

**Figure 3:**
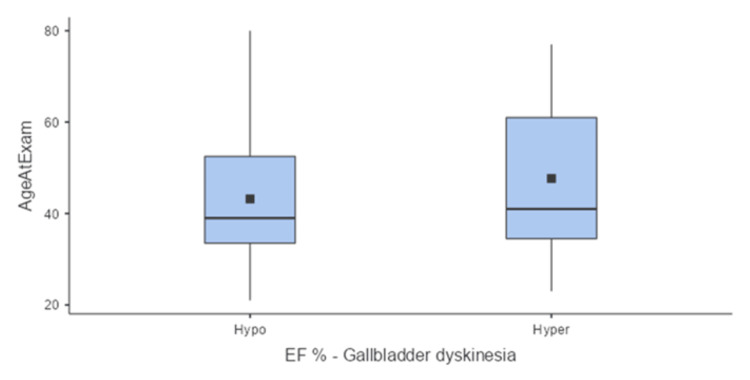
Comparison between the two groups according to age. Hypo: hypofunctional gallbladder; Hyper: hyperfunctional gallbladder; EF%: ejection fraction

**Figure 4:**
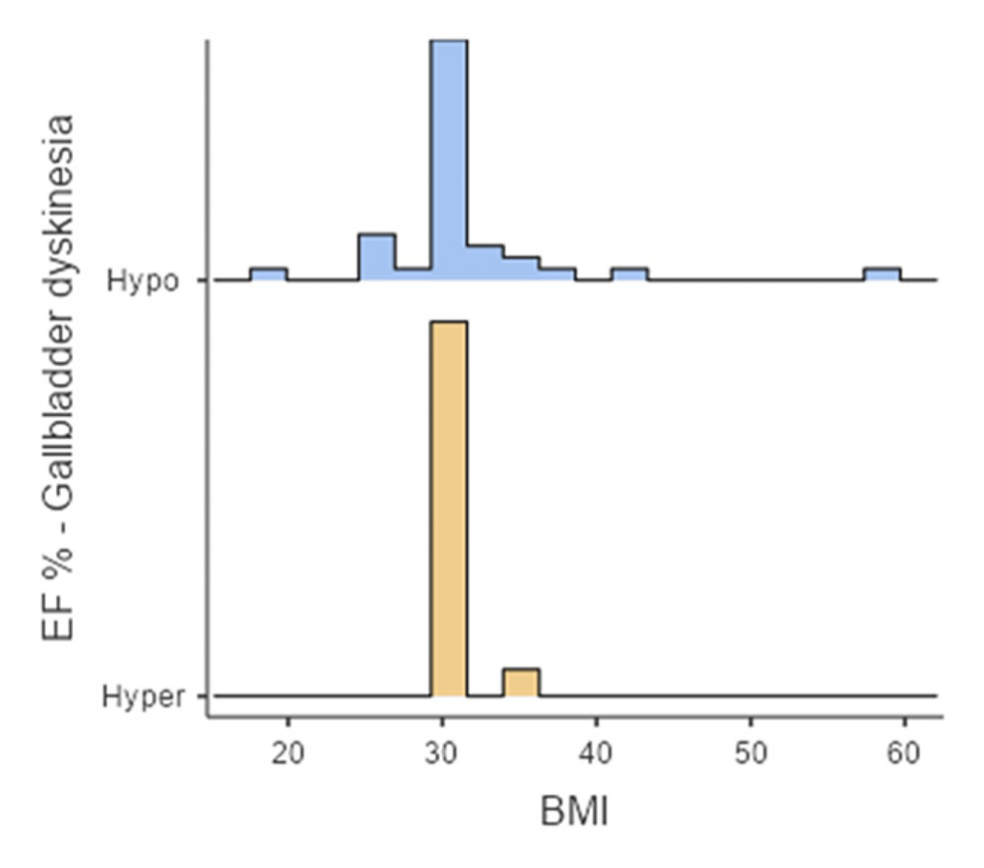
Comparison between the two groups according to BMI. Hypo: hypofunctional gallbladder; Hyper: hyperfunctional gallbladder; EF%: ejection fraction

Patients were then divided into two groups: the first group was diagnosed with hypofunctional gallbladder (EF<35%), and this group included 36 patients. Of them, 30 patients were females (83.8%), and six patients were males (16.6%) (Figure 2), with a median age of 39 (interquartile range (IQR) 21) (Figure 3) and mean BMI of 31.1 (± 9.17) (Figure 4).

All patients presented as outpatients with biliary-type pain that builds up to a steady level and lasts 30 minutes or longer, occurring at different intervals, related to fatty food administration, and severe enough to interrupt daily activities. Thirty-five patients underwent USS of the abdomen to assess gallbladder and bile ducts that came negative. Only one patient had a distended gallbladder wall, measuring 19 mm in length. Additionally, LFTs including bilirubin, alanine transaminase (ALT), aspartate aminotransferase (AST), alkaline phosphatase (ALP), and gamma-glutamyltransferase (GGT) were done to all patients, 19 patients had mildly insignificantly deranged LFTs (maximum ALP 114, maximum GGT 130, and maximum bilirubin 39), and the rest of the patients had normal LFTs. Additionally, 28 patients had their serum amylase done, and four patients had mildly deranged amylase (maximum value 168), which is not significant in diagnosing pancreatitis. The mean EF% calculated by the HIDA scan was 17.9% (± 7.38%).

In addition to bladder dyskinesia symptoms, those patients had associated symptoms including reflux symptoms because of gastrooesophageal reflux disease (GERD) or small hiatal hernia (six patients 16.6%), bloating and change in bowel habits because of irritable bowel disease (five patients, 13.8%), and both (only one patient, 2.7%).

**Figure 5:**
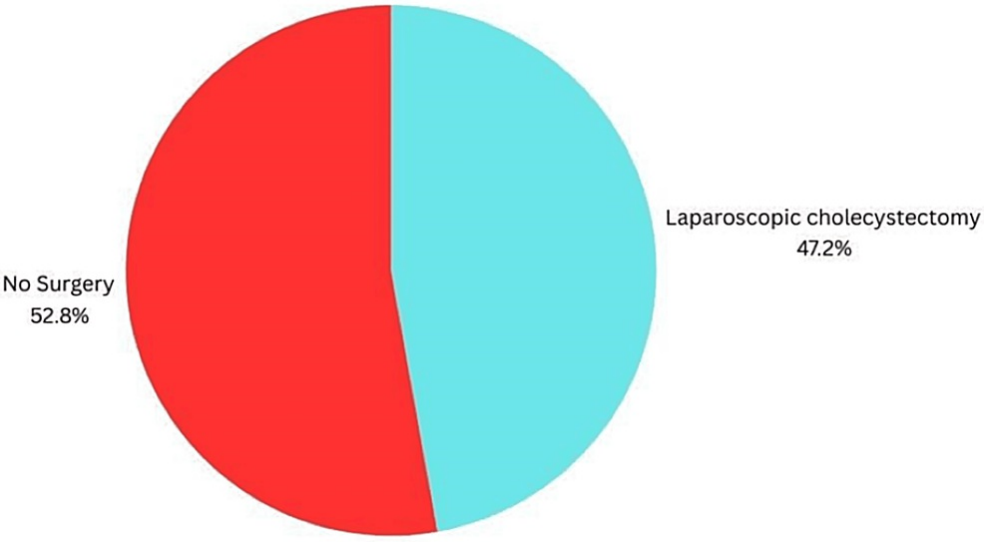
Pie chart showing the percentage of surgery vs no surgery in the low EF% group. EF: ejection fraction

Among patients diagnosed with hypofunctional gallbladder (EF <35%), 17 patients (47%) underwent laparoscopic cholecystectomy (Figure 5). Intraoperative findings were uneventful in 12 (70.5%) patients; however, in three (17.6%) patients, there were omental adhesions with the gallbladder, and in only one patient (5.8%) there was a capsular tear bleed that was controlled with diathermy. Postoperative histopathology showed no gallstones in all 17 patients who underwent surgery (0%).

**Figure 6:**
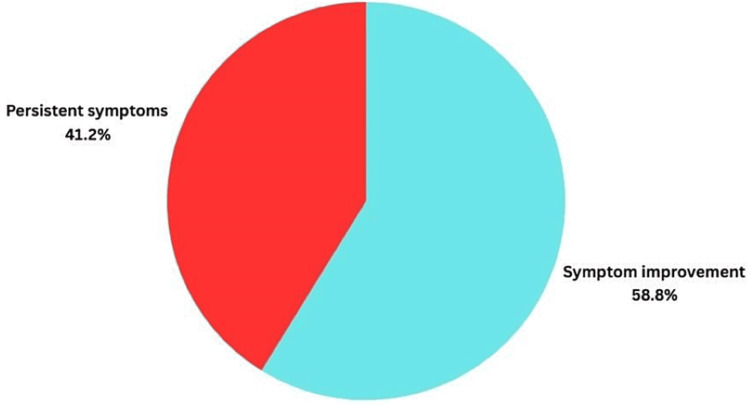
Pie chart showing the percentage of symptom improvement vs no improvement in the low EF% group. EF: ejection fraction

Symptom improvement after laparoscopic cholecystectomy occurred in 10 patients (58.8%) (Figure 6). The lack of symptom improvement in the other seven patients was attributed to the presence of either totally different diagnosis or concomitant disease such as slow colonic transit/(IBS-C) (two patients), sphincter of Oddi dysfunction or gastric ulcer (one patient), erosive reflux (one patient), fatty liver changes (two patients), and unknown cause patient (one patient).

Conversely, 19 patients were diagnosed with hypofunctioning gallbladder and did not undergo surgery, and the reasons behind this are loss to follow-up (two patients leaving 17 patients), patient refusal (eight patients), GERD symptomatology or possible irritable bowel syndrome (three patients), surgeon refusal because of lack of increased symptoms on the administration of CCK during HIDA scan (one patient) or because of non-intrusive symptoms (two patients), one patient had mild oesophagitis on oesophagogastroduodenoscopy (OGD), one patient still awaits surgery, one patient awaiting referral to upper GI (UGI) surgeon.

Alternatively, these patients received non-surgical treatment in the form of a gluten-free diet (two patients), other dietary modifications such as low fermentable oligosaccharides, disaccharides, monosaccharides, and polyols (FODMAP) diet and low-fat diet (five patients), proton pump inhibitors (PPI) (six patients), mebeverine (two patients), hyoscine butylbromide (two patients), and only analgesia (one patient).

Out of these 17 patients who received non-surgical treatment, symptom improvement occurred in seven patients (41.1%).

Conversely, the second group had the diagnosis of hyperfunctional gallbladder (EF>80%), and this group included 15 patients. Of them, 13 patients were females (86.66%), and two patients were males (13.33%) with a median age of 41 (IQR 24) (Table 1).

**Table 1:** Comparison between two groups according to demographic data. IQR: interquartile range

Dysfunctional gallbladder	Hypofunctional	Hyperfunctional
Number	36	15
Female:male (number/%)	30:6 (83.8%)	13:2 (86.66%)
Age (median/IQR)	39 (21)	41 (24)

Each patient experienced biliary-like discomfort that gradually intensified to a sustained level lasting at least half an hour, happening at various intervals, triggered by the consumption of fatty foods, and severe enough to disrupt their daily routines.

Among them, 12 patients underwent USS abdomen to assess gallbladder and bile ducts that came normal in 11 patients, three patients did not get a USS, and only one patient had abnormal US findings in the form of a small polyp measuring 4.5 mm in the posterior wall with no obvious gallstone seen (Table 2).

**Table 2:** Comparison between two groups according to US findings. US: ultrasound

Dysfunctional gallbladder	Hypofunctional	Hyperfunctional
Normal US (number)	34	11
Abnormal US (number)	1	1
Abnormal findings	Small calculi within the lumen	Small polyp measuring 4.5 mm

Liver function tests (LFTs) including bilirubin, ALT, AST, ALP, and GGT were done on 12 patients, 10 patients had normal LFTs, while two patients had mildly insignificantly deranged LFTs, and the remaining three patients did not have their LFTs tested. Additionally, 12 patients had their serum amylase done, 11 patients had normal serum amylase, one patient had amylase of 200 at the time of the HIDA scan, and the remaining three patients had no amylase tested. The mean EF% calculated by HIDA scan among this group was 88.5% (± 6.8%) (Table 3).

**Table 3:** Comparison between two groups according to LFTs and serum amylase. LFTs: liver function tests

Dysfunctional gallbladder	Hypofunctional	Hyperfunctional
Normal LFTs	17	10
Abnormal LFTs	19 (mildly insignificantly deranged)	2 (mildly insignificantly deranged)
Normal amylase	24	11
Abnormal amylase (value)	4 (maximum value 168)	1 (200; nonspecific)

The associated symptoms in these patients included GERD or small hiatal hernia (two patients 13.33%), irritable bowel disease (one patient 6.66%), one patient had suspected gastroparesis, and another patient had suspected celiac disease or sphincter of Oddi syndrome (Table 4).

**Table 4:** Comparison between two groups according to associated diseases. GERD: gastrooesophageal reflux disease; IBS: irritable bowel syndrome

Dysfunctional gallbladder	Hypofunctional	Hyperfunctional
GERD (number)	7	2
IBS (number)	6	1
Gastroparesis (number)	0	1
Celiac disease (number)	0	1

Among patients who were diagnosed with hyperfunctional gallbladder (EF >80%), two patients (13.33%) underwent laparoscopic cholecystectomy. Intraoperative findings were uneventful in one patient (50%) patients, and the other patient had omental adhesions with the gallbladder. Symptom improvement after laparoscopic cholecystectomy occurred in both patients (100%) (Table 5).

**Table 5:** Comparison between two groups according to laparoscopic cholecystectomy. IO: intraoperative; PO: postoperative

Dysfunctional gallbladder	Hypofunctional	Hyperfunctional
Number of patients underwent surgery	17	2
Abnormal intraoperative findings (number)	3	1
Description of findings		
Omental adhesions	2	1
Capsular tear bleed	1	0
Symptom improvement (number/%)	10 (58.8%)	2 (100%)
PO histopathological evidence of gallstones (%)	0%	0%

Conversely, 13 patients were diagnosed with hyperfunctioning gallbladder and did not undergo surgery, the possible reasons behind this were Barrett’s oesophagus and non-erosive gastritis, GERD, IBS, endometriosis and constipation, hiatal hernia, previous history of neuroendocrine tumour of the small bowel, nonspecific dyspepsia, fatty liver/probable ovarian cyst, and suspected gastroparesis.

Alternatively, these patients received non-surgical treatment in the form of amitriptyline and famotidine, a low-fat and low-carbohydrate diet, analgesia, and PPI. Unfortunately, only one patient had symptomatic relief.

Reported symptom improvement occurred in one patient, and the rest of the patients were either lost to follow-up or reported no symptom improvement (Table 6).

**Table 6:** Comparison between two groups according to symptom improvement.

Dysfunctional gallbladder	Hypofunctional	Hyperfunctional
Patients who had no surgery (number)	19	13
Patients who had medical treatment (number)	17	13
Symptom improvement after medical treatment	7 (41%)	1 (7.69%)

## Discussion

BD is characterised by biliary-type abdominal pain, absence of gallstones on the US, and reduced GBEF on a CCK-HIDA scan. The Rome IV criteria support the use of reduced GBEF as indicative of BD [2]. Laparoscopic cholecystectomy is the most common treatment for BD, although high-quality data supporting this practice are lacking [1]. Nonoperative medical treatments such as cisapride, erythromycin, nitric oxide, and cholinergic agonists are suggested alternatives to surgery [7]. Patients meeting diagnostic criteria for BD should be considered for cholecystectomy, with most experiencing symptom relief post-surgery [17]. The complexities of diagnosis and treatment, along with patient and provider preferences, hinder the conduct of randomised controlled trials (RCTs) for clearer evidence-based management of BD [18].

Despite the retrospective nature of our studies evaluating cholecystectomy for symptomatic gallbladder diseases, most of the available literature is retrospective and consistently indicates the effectiveness of cholecystectomy in improving symptoms, which is in line with our study results. Studies show high-resolution rates of biliary pain post-cholecystectomy, ranging from 66 to 100% [19], although our study shows only 58.8% symptom improvement following surgery. Additionally, laparoscopic cholecystectomy has been shown to have low rates of mortality and morbidity, making it a safe procedure with favourable outcomes [20].

One limitation of this study is the patients' follow-up. Some of the patients were lost to follow-up following surgery or even after receiving medical treatment. As a result, whether the modality of treatment has helped in symptom improvement or not, is not clear in these patients.

Two RCTs addressed gallbladder dyskinesia. The first one, the Yap trial, focused on evaluating 21 patients with biliary pain and reduced GBEF, comparing surgery to observation, showing superior results in the surgical arm but criticised for lacking an active nonoperative treatment arm [21]. In contrast, the other trial, the Richmond trial, aimed to address this by comparing surgery to active nonoperative therapy with amitriptyline, but it faced a high crossover rate from medical to surgical arms because of patient bias towards surgery as the preferred treatment [22]. Patients who underwent surgery in the Richmond trial experienced postoperative improvement using validated quality-of-life measures, highlighting the challenge of patient expectations and the absence of a definitive medical treatment standard influencing the preference for surgery [23].

A double-blinded RCT comparing laparoscopic cholecystectomy with medical treatments would indeed provide compelling evidence of the benefits of surgery for BD symptoms. Such a study would involve randomising patients to receive either intervention without their knowledge, ensuring unbiased results.

Standardised criteria for preoperative BD diagnosis and postoperative outcome measures would enhance the study's validity. Previous research has highlighted the importance of assessing the effectiveness and cost-effectiveness of surgical interventions such as cholecystectomy for gallstone-related conditions, emphasising the need for rigorous trials to guide clinical decision-making [21,23].

## Conclusions

We concluded that laparoscopic cholecystectomy is deemed a safe and effective option for symptom improvement in case of dysfunctional gallbladder, either in hypo- or hyperfunctional gallbladder despite the retrospective nature of our study. Medical treatment can act as an alternative to surgery in case of a patient's refusal, being unfit for surgery, or a consultant's opinion. Further RCTs are needed to compare the effectiveness of surgical management versus medical treatment in improving symptoms of BD.
